# Nursing ethical dimensions of euthanasia and medically assisted suicide for older people in need of long-term care

**DOI:** 10.3389/fpsyt.2025.1589487

**Published:** 2025-05-14

**Authors:** Anna-Henrikje Seidlein, Annette Riedel, Thomas Heidenreich, Karen Klotz

**Affiliations:** ^1^ Institute of Ethics and History of Medicine, University Medicine Greifswald, Greifswald, Germany; ^2^ Faculty of Social Work, Education and Nursing Sciences, Esslingen University of Applied Sciences, Esslingen, Germany

**Keywords:** old age, euthanasia, medically assisted suicide, long-term care, nursing care, nursing ethics, loneliness, vulnerability

## Abstract

Euthanasia and medically assisted suicide (E/MAS) are (healthcare-)practices intended to cause a patient’s death according to their wish and will. This article addresses the specific *ethical* questions that arise in the context of E/MAS regarding older adults in need of long-term care (LTC) from a nursing ethics perspective. Older people in need of LTC are particularly vulnerable due to a combination of health-related, social, and economic factors. Multiple chronic diseases, age-related frailty and the subsequent need for LTC, for example, may contribute to an increased risk of social isolation, loneliness and hopelessness. The boundaries between “normal” age-related changes and changes that are deemed to be pathological also become blurred. The complex interplay of these factors results in the particular needs of older adults who rely on nursing care. Consequently, specific ethical issues arise that are unique in terms of their quality and quantity – also regarding E/MAS. We present a common scenario: a fall with a subsequent fracture, as a turning point in the LTC arrangement. The consequences of such an adverse event (limited mobility, risk of further falls) compromise the security of care at home, raising the (potential) need for transfer to a LTC facility. This (potential) move represents a major disruption, is experienced as a crisis, and marks a transition for the older adult in need of LTC. We highlight the complex interplay between aging, health-care dependency, personal values and the resulting wish/request for E/MAS. We discuss how the professional self-perception and ethical values of the nursing profession in the home care setting determine the treatment pathway for older peoples’ request for E/MAS.

## Introduction

Euthanasia and medically assisted suicide (E/MAS) are two distinct practices of voluntary assisted dying having in common that they both intentionally and directly bring about the death of a person (resp. patient). While in the former, a healthcare professional actively administers the lethal substance, in the latter, the patient takes the lethal dose of the drug themselves.

In Germany, medically assisted suicide is legal since the Federal Constitutional Court declared §217 of the German Criminal Code to be void in 2020; Euthanasia remains illegal.

There are many compelling reasons to examine the ethical complexities of E/MAS through the lens of nursing, as nurses play a pivotal role in patient care, advocacy, and ethical decision-making. Although their skills and expertise in this respect are becoming increasingly visible in academic discourse in Germany (c.f. 1, 2), they continue to be given too little consideration in political and public debates.

In this perspective article, we focus on the most pressing ethical issues arising in the context of nursing care for patients requesting E/MAS and illustrate the ethical implications in view of their professional self-image and values, based on a case example. This focus should by no means neglect the fact that E/MAS is an *interprofessional* practice in which a trusting relationship and teamwork with clear roles between nurses, physicians and other healthcare professionals is crucial. Special attention must be paid to “Hierarchy, communication and group processes” (3) to ensure ethical practice, quality of care and healthy staff.

*Firstly*, nurses play a central role in the lives of people in need of care – regardless of the setting or patient population (4). On the one hand, this is simply due to quantitative aspects: Particularly in long-term care (LTC), nurses serve as primary points of contact on a daily basis and over extended periods of time. In addition, the daily encounter is very immediate and characterized by physical proximity, touch (5), and actions that concern the intimate sphere as well as conversations about potentially shameful topics. On the other hand, nurses hold their key position not only for quantitative reasons but also due to qualitative factors: Nursing as a profession is perceived as trustworthy by those in need of care and a deep sense of trust often develops in the nurse-patient relationship (6). Nursing care is grounded in interaction and relationship (7). Both are a prerequisite for good care, developed intentionally, and modified constantly. Interpersonal relationships and the ethical bonds and responsibilities they entail are “at the heart of the moral realm of nursing practice” (8, p. xiv). Nursing care does not allow for rigid daily routines; nevertheless, it demands the consideration and harmonization of subjective, individual needs in conjunction with objective nursing and medical requirements (9). This professional negotiation is based on the respective relationship leading to extensive knowledge about peoples’ values and expectations. Nurses “cultivate ethical knowledge of at least two forms: (1) relational knowledge; and (2) embodied knowledge” (10, p. 20). Consequently, nurses are often the first to whom individuals confide their thoughts and emotions – including requests for E/MAS (11, 12).

*Secondly*, nurses have a specific professional ethos which distinguishes them from other healthcare professions, particularly medicine. Although there are overlaps and similarities, a nursing ethics perspective is distinct from medical ethics, reflecting the professions’ foundations, historical development, and professional mission (13). The values to which nurses commit themselves are outlined in the International Council of Nurses’ (ICN) Code of Ethics for Nurses (14), which is guided by human rights. The key values and moral norms of the nursing profession include caring, compassion, empathy, and advocacy for patients’ interests (14). One of the four “fundamental nursing responsibilities” is “to alleviate suffering and promote a dignified death” (14, p. 2). Nursing Ethics is – especially at its roots– “relationally based” (13, p. 33, 246). This is reflected not only in the professional ethos of nurses, but also the ethical theories that are predominantly referred to in nursing ethics (e.g. care ethics). Nursing *care* is, thus, both a social practice (descriptive) and a moral value (normative) (15). Promoting a dignified death is a term that lends itself to many different interpretations: While, on the one hand, it relates directly to palliative care interventions, it may, on the other hand, encompass dealing with requests for E/MAS.

To summarize, it can be stated that nurses play a direct role in dealing with requests for E/MAS owing to their unique role and mission within healthcare. Furthermore, from the normative standpoint of nurses’ professional ethos, it aligns with their role and responsibility to engage with this topic not only in terms of care for older adults requiring LTC – which are the focus of the following reflections – but also regarding their own well-being and self-care.

## A paradigmatic case

Mrs. Elisabeth R. is 82 years old, has been widowed for a few months and lives alone at home. She is in a good cognitive condition and takes care of herself, however, the increasing loss of her eyesight hampers her. A fall when going to the toilet one night, results in a fractured femur and endangers her previous way of life. Her mobility is now limited, she needs support in handling everyday life, and is at risk of further falls. The anesthesia required to repair the fracture induced a delirium, leaving a lasting effect on her cognitive abilities. Elisabeth’s daughter Anna has taken on the responsibility of caring for her mother, while trying to juggle her management position at work and family life, placing her under immense pressure. Although she receives support from a home nursing service, she feels that her own career and relationship are being affected negatively, as caregiving responsibilities increasingly take over her life. Moreover, the home nursing service only helps selectively and is neither a permanent solution in terms of time nor money. Elisabeth’s daughter perceives the home care arrangement as increasingly difficult and is concerned about her mother’s safety and quality of care. She, therefore, suggests moving her mother to a LTC facility, but Elisabeth insists on staying at home. She argues that a nursing home is meant for those suffering from dementia and frailty, a place where she would lose her quality of life. She fears that she will ‘lose her mind,’ lose control over her life, and, as a result, her dignity. Following some discussion and persuasion, Elizabeth finally agrees, and Anna arranges the move to a LTC facility.

Since moving to the nursing home, Elisabeth has become increasingly introverted. Conversations during her daughter’s visits become progressively one-sided and the nurses note that Elisabeth rarely participates in joint activities. She repeatedly expresses the desire “to no longer be a burden,” and one day, even asks the nurse “to end her suffering.” The nurse is shocked and uncertain how to react. She had never encountered a situation like this before and is not sure how to respond appropriately. Therefore, she pretends not to hear the comment. After leaving the room, she takes the first opportunity to call Elisabeth’s daughter who responds: “Mom has been saying this a few times lately, but I thought she couldn’t really mean it. This sadness and hopelessness is just normal, considering the profound changes in her life”.

## Ethical tensions from a nursing ethics perspective

The specific professional focus of nurses and nursing ethics draw special attention to a number of aspects that arise from empirical data on older adults in need of LTC and normative claims of nurses’ professional ethics. They are important for the development and handling of requests for E/MAS not only in Elisabeth’s case but also in similar cases.

All individuals involved in this case have a specific *vulnerability* that is temporarily increased by E/MAS either by formulating the request (Elisabeth), receiving the request (nurse) or through being informed about the request (Anna). These intertwining vulnerabilities in relationships and the situational context matter, because they give concrete meaning to abstract ethical principles, which cannot be understood on their own or applied rigidly.

Being dependent is an unavoidable element of human existence due to the central role of mutual relationships as stressed within nursing ethics. Moreover, dependence implies asymmetry (16). Consequently, an individualistic concept of autonomy focusing solely on rationality and independence, as well as equating autonomy with the ability to consent, cannot be applied, as it does not reflect real life. Instead, relational autonomy assumes that the ability to make decisions must not be understood in isolation but in the context of social relationships and structures (17, 18). The focus here is on reflection and taking responsibility for one’s actions within different kinds of relationship (e.g. nursing relationship, mother-child relationship) and in view of the individual’s situation-specific vulnerabilities.

The entanglement of vulnerabilities results from different sources of vulnerability concerning the older person in need of LTC and the interconnection of the specific situational vulnerability of the other different parties involved. In the case scenario, Elisabeth’s vulnerability is increased by physical health impairments, the loss of a loved one, her personal environment, and her transition into a nursing home. The majority of (older) adults in need of care wish to avoid moving into an inpatient LTC facility (19). This move is often not perceived as a voluntary choice but rather as a result of being without alternative options, leaving one feeling at someone else’s mercy (20). Older adults may fear a loss of autonomy (21) and this fear frequently becomes a lived reality. Ultimately, this conflicts with the ethical goal of self-determination (22). Thus, moving into a LTC facility is a major turning point; it represents a transition that requires a process of adjustment (23). Older people in LTC are highly vulnerable, at least temporarily, due to the transition being a critical life event. In some cases, this vulnerability can become permanent, for example, due to an increased risk of human rights violations in nursing homes (24, 25). By expressing her desire for E/MAS, Elisabeth is once again particularly vulnerable, as she cannot be sure of the open and empathic reactions of those around her. However, little has been discovered about this side of the ‘triad’ (older person – relatives/family – nurse) and their lived experiences so far (26, 27) at least not regarding the setting of nursing homes.

The admission of a parent to a LTC facility is often accompanied by a mix of conflicting emotions, such as relief and guilt, as well as being blamed by others (28, 29). This constellation heightens the vulnerability of Anna, the daughter and informal caregiver, who finds herself in a similar position.

Finally, the nurse’s vulnerability is increased by the moral complexity. The latter refers to situations in which moral issues are characterized by conflicting values, principles, or duties – both at the intra- and interindividual level. On the epistemic level (i.e. concerning the knowledge of those affected), this may be the case in the presence of a moral dilemma or moral uncertainty about the ‘best’ course of action – both leading to moral discomfort (30). As a result, the ability to act is hampered, one’s conscience challenged and moral distress provoked (31).

This is particularly true given the fact that the nurse has neither sufficient knowledge on the topic of requests for E/MAS, access to institutional guidelines to support any actions considered nor the opportunity for targeted ethical reflection within the team. Despite the fact that requests for E/MAS in older people are not uncommon (32), nurses do not always feel prepared and competent to deal with them (33, 34). If the nurse realizes this and reflects on it in view of their ethical obligation, their vulnerability can be further increased by feeling unable to meet nurses’ professional ethical standards.

## Discussion

Through the lens of nursing ethics, with a focus on relationality and vulnerability, it follows for the discussion on E/MAS that nurses hold a pivotal position, where their immediate reaction is central to the experience (e.g. being taken seriously) of older people requesting E/MAS. Their professional self-perception and personal as well as professional moral values can determine the treatment pathway.

Nevertheless, if not precisely for this reason, conscientious objection due to moral and/or religious reasons must remain possible for nurses, relying on their team without polarization and dogmatism (see e.g. 35).

Yet there are remarkable knowledge gaps concerning E/MAS (36). Nurses should be prepared through specific educational *content* – such as legal and ethical aspects (37) as well as palliative care and advanced care planning (38) – and *methods* (such as simulation-based learning on how to deal with requests for E/MAS) (39). In addition, nurses must be aware that the move into a nursing home and associated changes to the personal identity of the older adult may cause a wish for E/MAS. Hence, nursing interventions aiming at good transitional care [e.g. improving emotional well-being, personalized care (40, 41)] are important measures for suicide prevention. These should naturally be supported by a framework for the early detection of depressive symptoms.

Nursing home residents appreciate an open communication about the end of life and their preferences and needs (42). This also entails that nurses approach every request for E/MAS with diligence and respond to it without any preconceptions. Ageism may play a significant role in how requests for E/MAS from older people are perceived and handled, potentially leading to a lack of attention to their autonomy, unaddressed social or medical needs, and the ethical complexities involved. Hence, nurses must not allow themselves to be influenced by ageism that can bias their understanding. The prerequisite is that they become aware of and reflect on them critically.

Relational autonomy, which emphasizes the interconnectedness of individuals and the influence of social and care relationships on decision-making, plays a crucial role in addressing requests for E/MAS by ensuring that such decisions are understood within the broader context of the person’s vulnerabilities, support systems, and care environment. Thus, “A satisfactory solution based on relational autonomy must incorporate patients’ competence (apart from decisional capacity), authenticity (their true desires or beliefs) and the involvement level of their significant others” (43, p.1).

Moreover, as the case of Elisabeth impressively shows, the close connection between the wish for E/MAS and existential questions and crises must be taken into account (see also discourse on ‘existential suffering’, e.g. 44, 45). Although E/MAS is by its very nature an existential issue, existential questions are not always to be understood as a request for E/MAS. Karl Jaspers’ concept of the *boundary situation* (46) – experiences like death, suffering, and existential conflict that confront individuals with the limits of existence – offers a profound philosophical framework for understanding the inner struggles and meaning-making processes of both patients facing decisions around medically assisted dying and their caretakers. It is important to note that existential suffering is highly complex and often ambiguous, and as a result demands a high degree of sensitivity to each individual situation (44, 47). However, in any case, nurses are key actors in managing such situations (48).

Ultimately, E/MAS remains “complex (manifold) and puzzling (paradoxical)” (49, p. 373), calling for an interprofessional team effort that recognizes and reflect the varying ethical values, needs, and (asymmetric) relationships of all individuals involved.

## Data Availability

Data sharing is not applicable to this article as no new data were created or analyzed for the purpose of this publication.
